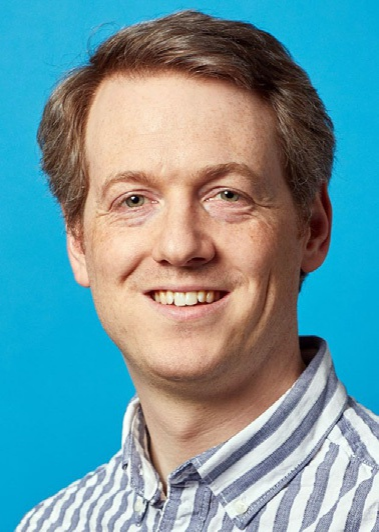# Functionalised N‐Heterocyclic Carbene Ligands in Bimetallic Architectures

**DOI:** 10.1002/chem.201905510

**Published:** 2020-03-18

**Authors:** Kieren J. Evans, Stephen M. Mansell

**Affiliations:** ^1^ Institute of Chemical Sciences Heriot-Watt University Edinburgh EH14 4AS UK

**Keywords:** bimetallic complexes, bridging ligands, ditopic ligands, metalation, N-heterocyclic carbenes

## Abstract

N‐Heterocyclic carbenes (NHCs) have become immensely successful ligands in coordination chemistry and homogeneous catalysis due to their strong terminal σ‐donor properties. However, by targeting NHC ligands with additional functionalisation, a new area of NHC coordination chemistry has developed that has enabled NHCs to be used to build up bimetallic and multimetallic architectures. This minireview covers the development of functionalised NHC ligands that incorporate additional donor sites in order to coordinate two or more metal atoms. This can be through the N‐atom of the NHC ring, through a donor group attached to the N‐atom or the carbon backbone, coordination of the π‐bond or an annulated π‐donor on the backbone, or through direct metalation of the backbone.

## Graphical abstract

**Bimetallic carbene complexes**: This minireview covers the use of N‐heterocyclic carbene (NHC) ligands in the formation of bimetallic architectures. NHCs are shown to bind one metal through the conventional C2 position and coordinate to another through donor atoms tethered to the NHC, through the N atom itself or through the NHC backbone. These bimetallic species are potentially useful models for decomposition reactions in homogeneous catalysis and organometallic chemistry.

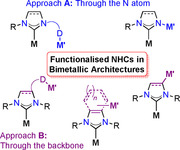

## Introduction

Bimetallic architectures combine two atoms of the same metal (homobimetallic) or two different metals (heterobimetallic) in order to generate more diverse properties and chemical possibilities than from using one metal alone. Bimetallic compositions have demonstrated improved properties and reactivity in the solid state and in heterogeneous catalysis,^1^ and the idea of using multiple metal atoms has also been successfully exploited in coordination chemistry,^2^ deprotonative metalation^3^ and homogeneous catalysis.^4^ With the utility of N‐heterocyclic carbenes (NHCs) now well and truly established in coordination chemistry and catalysis,^5^ more diverse designs of NHCs are now being explored to expand upon this area.^6^

**Figure 1:**
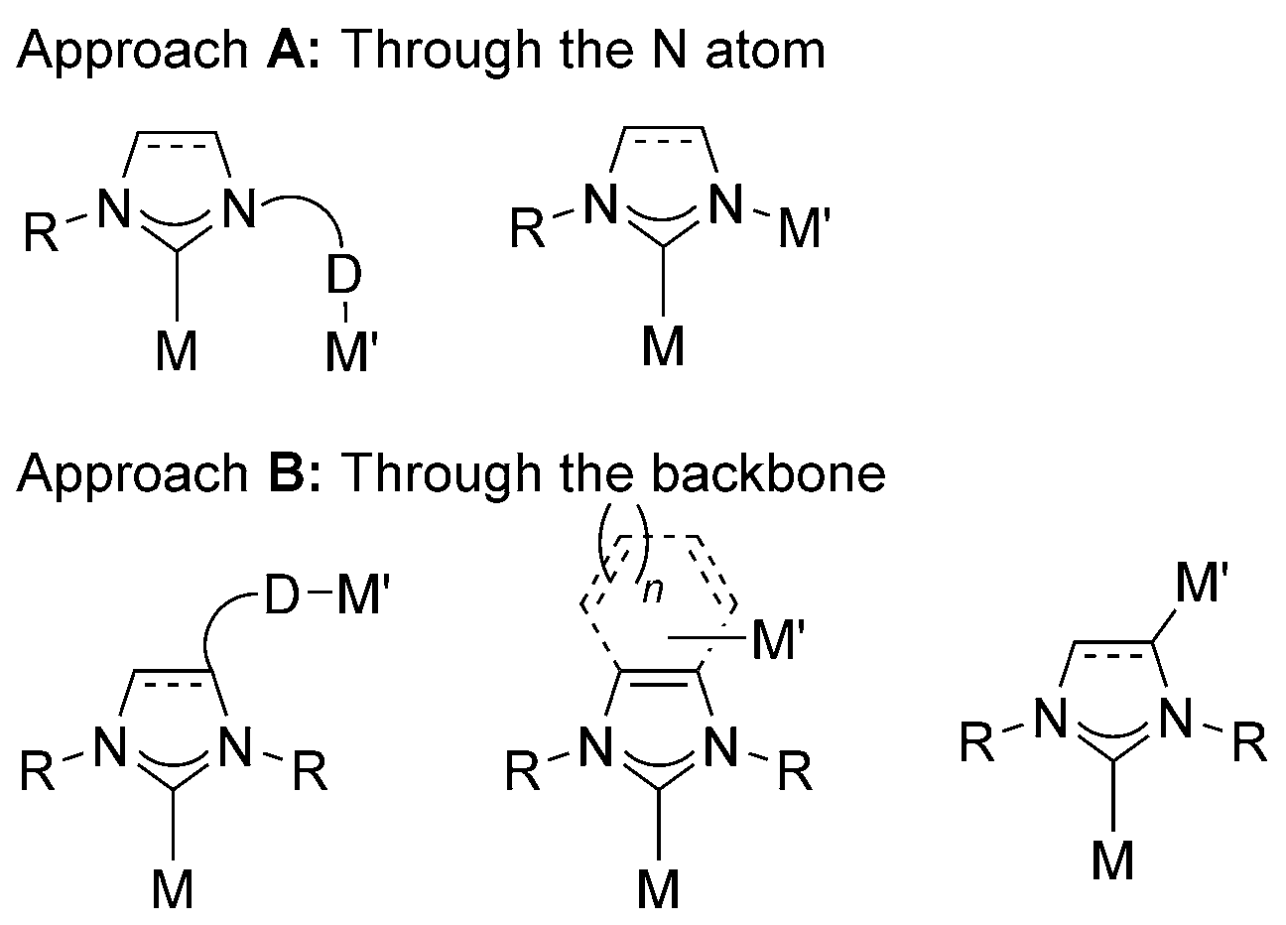
Approaches to functionalise an NHC ligand in order to form bimetallic architectures.

NHCs are strongly binding, terminal ligands,^7^ unlike their Sn analogues that often show bridging behaviour,^8^ so developing bimetallic complexes based on NHCs has required extra functionalisation of NHC ligands. This can be achieved through the addition of another donor connected to an N atom, or even directly through the N atom itself (Figure 1 A). The other location for introducing a second donor site is through the ligand backbone, by tethering a second donor group, through coordination to a π‐system or by direct metalation of the NHC backbone (Figure 1 B). In order to introduce two metal atoms, the resulting ligand must not chelate to the same metal atom. Recent reviews have covered separately the different ways NHCs can be functionalised, often focusing on chelating ligands rather than the formation of bimetallic architectures. These include NHCs tethered to an anionic donor,^9^ anionic carbenes,^10^ chiral NHC ligands with additional chelating groups,^11^ NHCs equipped with phosphine oxide substituents^12^ and NHCs with O‐donor and S‐donor substituents.^13^ This mini review will introduce the different ways that NHCs have been used to generate bimetallic architectures, but does not review ligands with multiple NHC donors,^14^ instead focusing on a representative selection of hybrid ligands^15^ with different donors that generate bimetallic architectures.

## N‐Functionalised With Donor Groups

### NHCs N‐substituted with Cp moieties

**Figure 2:**
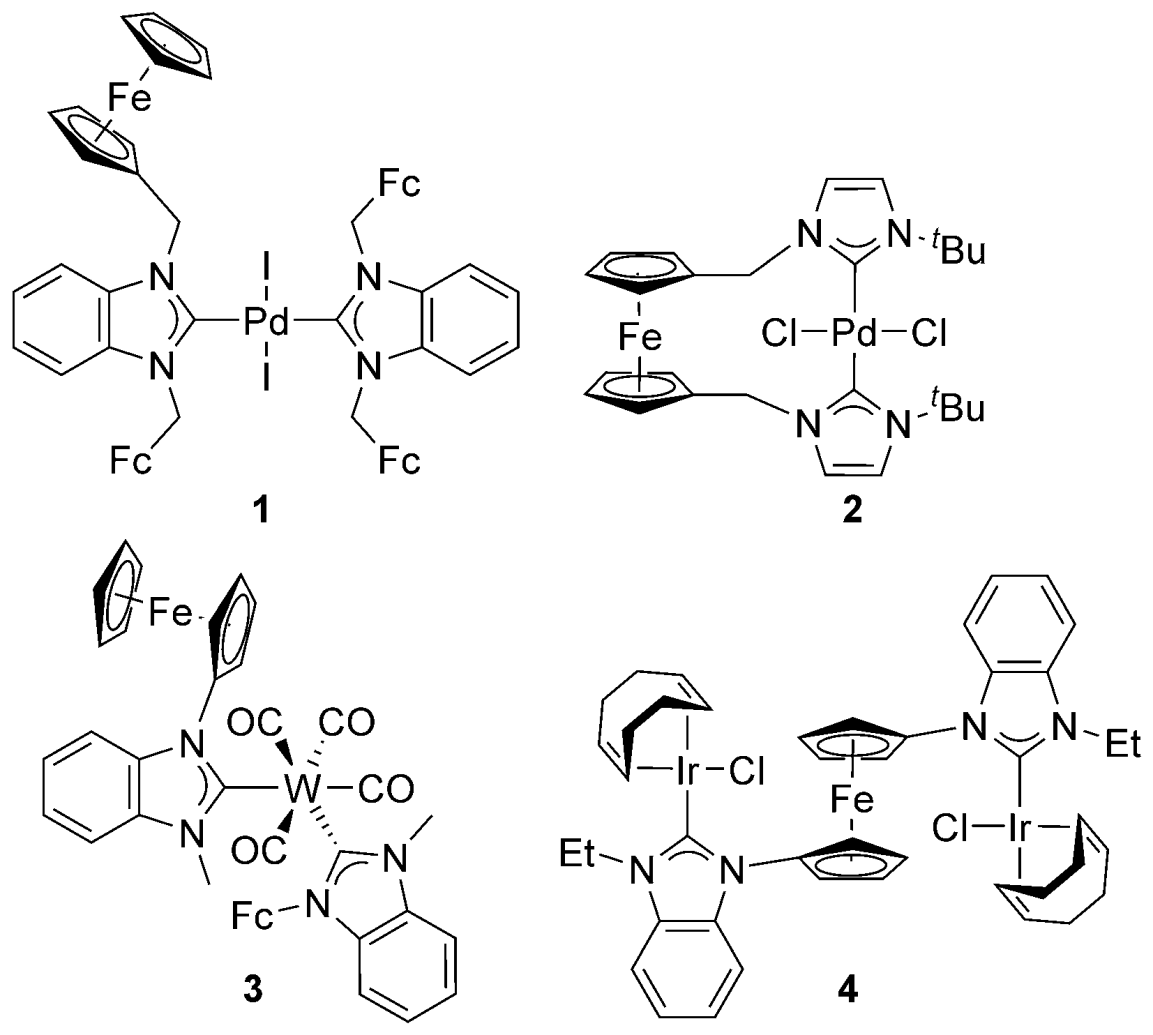
NHC complexes incorporating ferrocene (Fc).

Ligand systems featuring anionic Cp (Cp=η^5^‐C_5_H_5_) groups, and related donors such as indenyl (Ind) and fluorenyl (Flu), tethered to NHCs have been extensively studied.^16^ The focus has mainly been on the formation of chelating complexes, however, bimetallic complexes (and those with three or more metal atoms as well) have also been synthesised. With a single C atom separating the N atom and Cp, there are now a substantial number of complexes with the Cp group coordinating to a [FeCp] fragment forming an NHC with ferrocene as a substituent (e.g. **1**, Figure 2)^17^ or as part of a pincer framework (**2**).^18^ Ferrocenyl groups directly attached to the N atom are also well known (e.g. **3** and **4**).^19^ Carbenes that incorporate ferrocene have been the subject of a review.17c

**Scheme 1:**
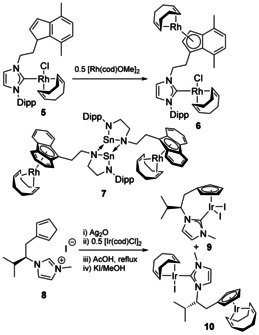
Bimetallic Group 9 complexes. Dipp=2,6‐diisopropylphenyl.

Aside from Fe coordinated to the Cp ligand, or derivative, examples are much rarer. In addition to tethered chelating indenyl‐NHC complexes with Rh and Ir,^20^ a dirhodium complex was also formed through metalation of the indene‐fragment with [Rh(cod)(μ‐OMe)]_2_ (Scheme 1, **6**; cod=1,5‐cyclooctadiene). The product features both square‐planar 16‐electron and half‐sandwich 18‐electron Rh geometries, which highlights the flexibility of this system and its ability to support multiple coordination modes. A dirhodium species was also formed using a tethered N‐heterocyclic stannylene (NHSn, **7**).^21^ Unlike with NHCs, NHSns often dimerise through dative N−Sn bonding,^22^ and this remained intact even in the presence of a Rh atom that could lead to a chelating complex. The resulting multimetallic complex showed η^5^‐binding of the Rh to the fluorenyl tethers, and coordination of cyclooctadiene. Bimetallic complex formation was also observed for Ir with a Cp‐tethered NHC ligand.^23^ Here, attempts to synthesise the half‐sandwich chelate complex **9** were hampered by the formation of the homobimetallic Ir species **10**, suggesting a lack of preference for the chelate even when controlling the stoichiometry to target the chelating complex.

**Scheme 2:**
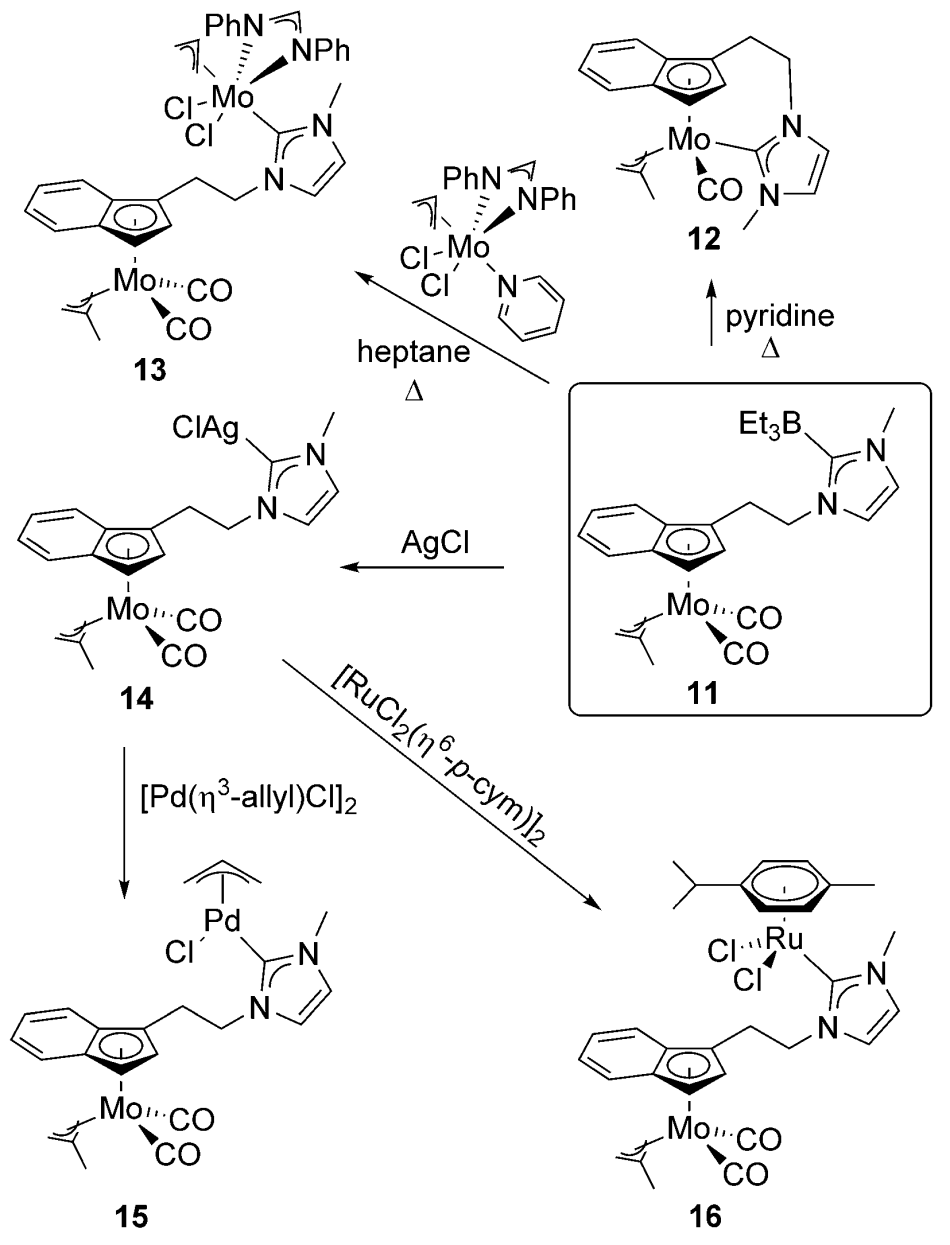
Bimetallic complexes with Mo. *p*‐cym=1‐Me‐4‐*i*PrC_6_H_4_.

The use of BEt_3_ as a carbene protecting group allowed the metalation of an indenyl‐NHC ligand to form a Mo complex with a pendent BEt_3_‐protected NHC (Scheme 2). From the monometallic species (**11**), both chelating monometallic and homobimetallic complexes were accessible by variation of the reaction conditions. Pyridine was found to remove the BEt_3_ protecting group and afford the chelated half‐sandwich complex **12**, whereas in order to achieve the homobimetallic species **13**, heptane was chosen as the solvent and a Mo precursor with a labile pyridine ligand was used, which was able to dissociate and cleave‐off the protecting group in situ.^24^ Extending this methodology to the late transition metals Ru and Pd was not successful, however, the reaction with AgCl gave the silver carbene complex **14** that was reacted with [Pd(allyl)Cl]_2_ and [RuCl_2_(*p*‐cymene)]_2_ to give heterobimetallic complexes **15** and **16**.^25^ Ag carbene complexes are widely used, and it is interesting to note that the silver route was essential to form the Group 8 and 10 complexes.

**Scheme 3:**
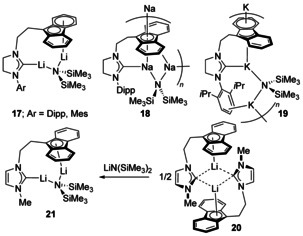
Homobimetallic tethered‐NHC complexes with alkali metals.

A series of homobimetallic fluorenyl‐tethered NHC complexes with Li, Na and K have recently been reported (**17**–**19**, Scheme 3).^26^ These saturated NHC complexes were synthesised from a spirocyclic precursor using a synergic reaction mixture of either LiPh, Li*n*Bu or MCH_2_Ph (M=Na, K) with an equimolar amount of the respective metal amide. All of the resulting compounds feature a bridging amide between the two metal centres that are centred on the fluorenyl‐NHC pocket. For Li, the species are molecular and are soluble in aromatic solvents, whereas for Na and K, the products are polymeric in nature and display lower solubility. A variety of different metal–arene coordination modes are present: η^6^, η^5^, η^2^ and η^4^. For the unsaturated tethered‐NHC ligand with N‐Me substitution, the synthesis could proceed via the neutral fluorene‐tethered NHC allowing the subsequent deprotonation to occur without metal amide present. This produced a unique example of a bridging NHC ligand situated between two Li atoms that are each coordinated η^5^ to a fluorenyl ring (**20**). Addition of LiN(SiMe_3_)_2_ in a second step could then be probed demonstrating the facile biding of metal amides by these species into the fluorenyl‐NHC pocket generating the unsaturated NHC complex **21**.26b

### N‐Donor

#### No linker

**Figure 3:**
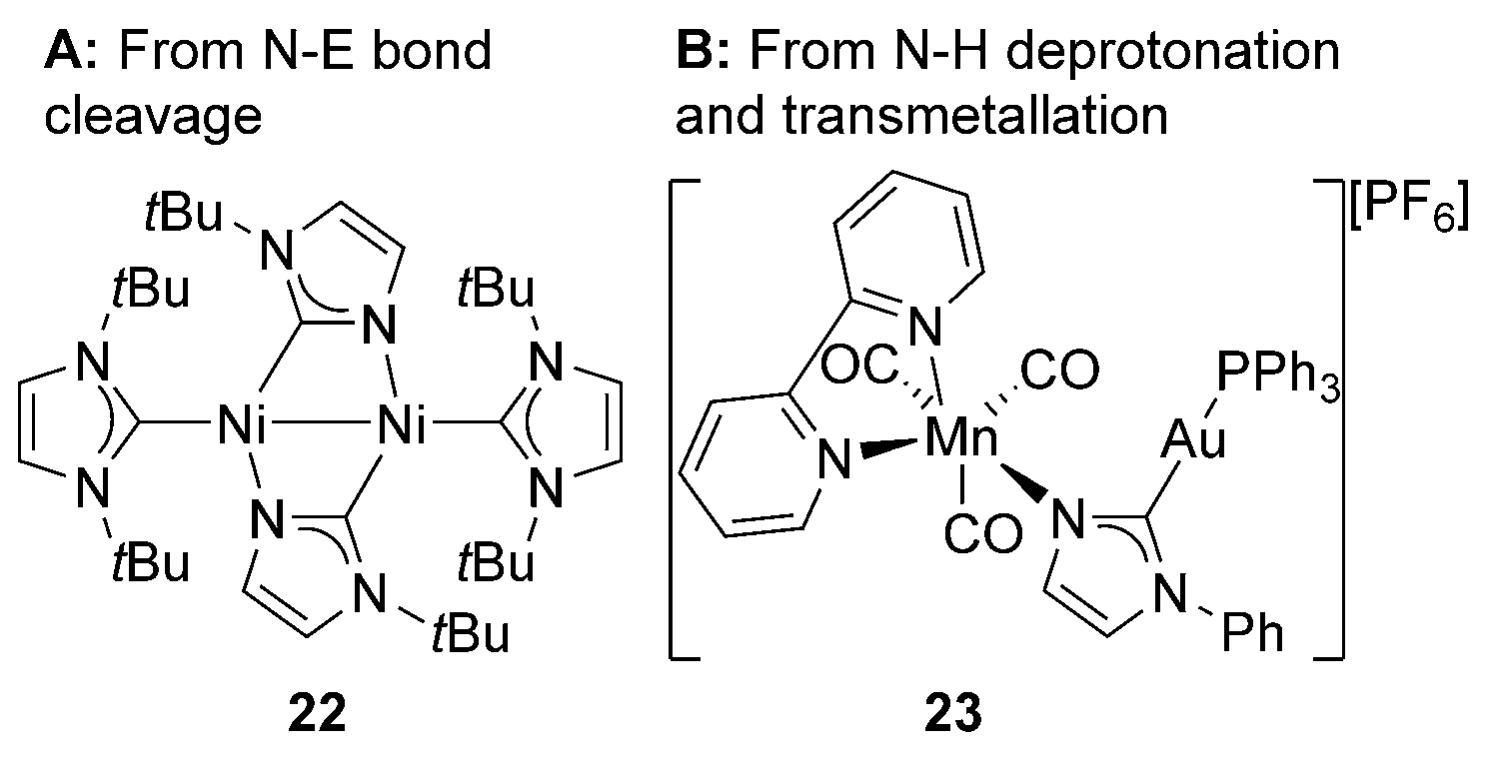
Bimetallic architectures with metal ions bound through the NHC C and N atoms.

Two standard routes are commonly used to bind metals to both the N and C atoms of an NHC: bond cleavage of the N‐substituent bond or deprotonation of an N−H substituted NHC (Figure 3). The homobimetallic Ni complex **22** was synthesised from the reaction of [Ni(1,5‐cod)_2_] with 1,3‐bis‐*tert*‐butylimidazol‐2‐ylidene in THF, which led initially to C−H oxidative addition of a Me group followed by N−C bond cleavage (with loss of isobutylene) and binding of the Ni atoms through the carbon and adjacent N atom.^27^ There are additional examples of C−N bond activation leading to multimetallic architectures,^28^ as well as N−P bond activation in N,N′‐diphosphanyl NHC ligands.^29^

**Scheme 4:**
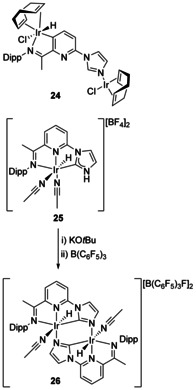
Formation of a bimetallic Ir complex.

Although most NHCs have substituents on both N atoms, protic NHCs have only one and thus have a reactive hydrogen substituent on the other N atom. This allows for new coordination modes with metalation possible at the carbene position or at the N atom.^30^ A Mn complex containing a protic NHC was deprotonated and coordinated to Au forming a heterobimetallic complex, although with the carbene C atom now binding to Au (Figure 3, **23**).^31^ Deprotonation of the NH on metal‐coordinated protic NHCs (followed by *trans*‐metalation where necessary) is now a general route to heterobimetallic complexes.^32^ One specific example is from the development of an NNC pincer ligand based on an iminopyridine tethered to a protic NHC (Scheme 4). Initial results led to the formation of a homobimetallic complex containing Ir coordinated to the N atom of the imidazole ring as well as between the imine group and the deprotonated pyridine ring (**24**), thus not containing an NHC at all. Different synthetic methodology led to the formation of the desired pincer ligand bound to Ir (**25**), and deprotonation of the N−H then led to the homobimetallic Ir complex **26**, described as containing bridging imidazolide donors.^33^

**Scheme 5:**
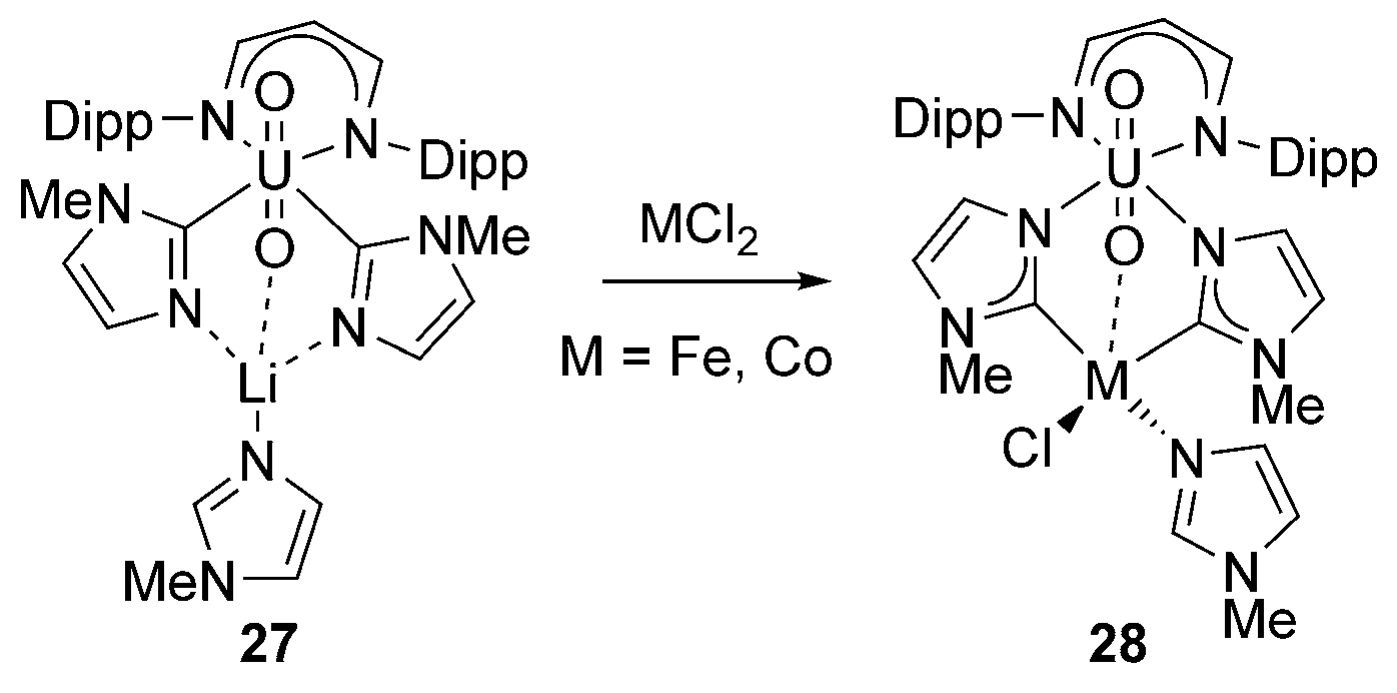
Uranyl/ transition‐metal bimetallic complexes.

A different synthetic method relied on the reaction of 2‐lithio‐1‐methylimidazole with a uranyl β‐diketiminato chloride complex that led to a heterobimetallic imidazolide complex with the C atoms bound to U and a Li cation bridging between the N atoms (Scheme 5, **27**). Upon reaction with CoCl_2_ and FeCl_2_, rearrangement was observed with the carbene C atom binding to the transition metal (TM, **28**).^34^

#### N‐Donor attached as N‐substituent

**Scheme 6:**
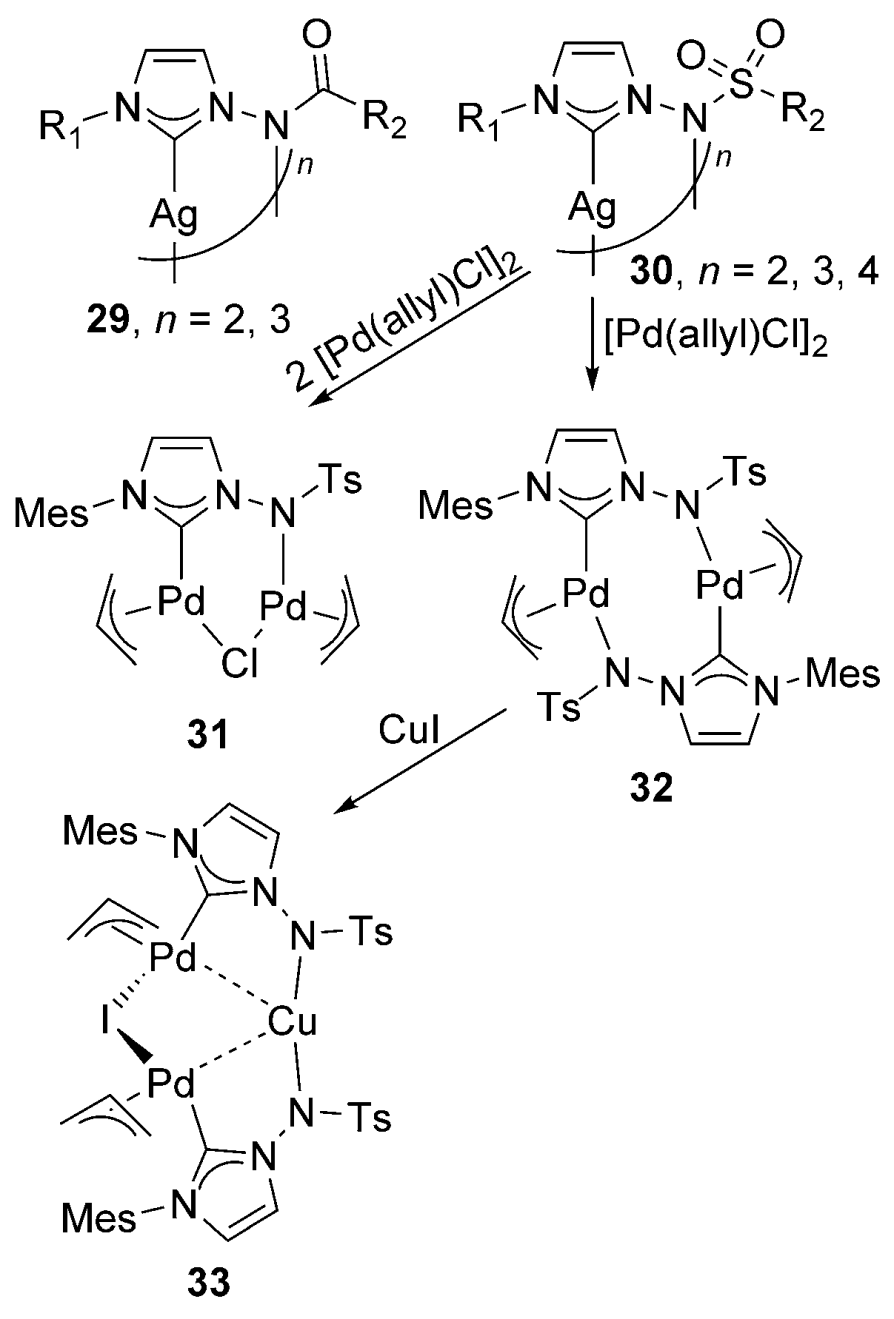
N‐Sulfonylimino and N‐acylimino complexes. Ts=SO_2_‐4‐MeC_6_H_4_.

N‐Sulfonylimino substituted NHCs were found to favour bridging interactions over chelation due to the highly strained nature of the chelate rings that would be formed.^35^ This is in contrast to similar N‐acylimino NHCs, which can chelate or bridge.^36^ For N‐sulfonylimino substituted NHCs coordinating to Ag ions (Scheme 6), cyclic trimeric structures were most commonly observed, with one tetramer found for the most sterically encumbered example (**30**). For the acyl analogues, dimers were more often observed (**29**).^36^ Transmetalation of Ag N‐sulfonyl NHCs with the Pd allyl chloride dimer (APC) led to the formation of homobimetallic Pd species **31** with two equivalents of APC, whereas one equivalent leads to a Pd dimer (**32**). The formation of both species relied on the bifunctionality of the NHC ligand and the close proximity of the two donor groups. The addition of CuI to the Pd dimer **32** forms a heterotrimetallic complex with two Pd and one Cu atom (**33**). This complex features Cu−Pd interactions that have been formalised as anionic Cu with a cationic Pd−I−Pd fragment.^35^ This arises from the differences between the neutral NHC and the anionic NTs donor, and with the Pd being softer in nature than Cu, has a preference for the NHC.

#### N‐Donors linked to the N‐atom

**Scheme 7:**
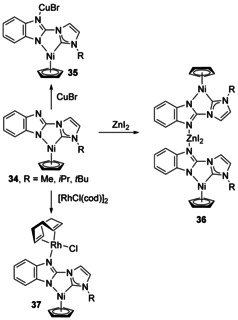
Bimetallic complexes based on a benzimidazolate‐NHC ligand.

Numerous examples of multimetallic complexes exist based on an additional N‐donor tethered to the NHC, so the examples presented will be necessarily selective. Additional pyridyl donors^37^ (or analogues such as bipyridine^38^ or phenanthroline^39^) bound to Cu or Ag are most commonly observed.^40^ Pd complexes with N‐pyridazine NHCs are covered later (Figure 24) given that they also feature backbone metalation. Very recently, heteronuclear complexes based on the benzimidazolate‐NHC ligand have been developed (Scheme 7).^41^ A [NiCp] fragment was first coordinated to the N,C chelate, which leaves the other N atom in the benzimidazolate ring open to coordination (**34**). Heteronuclear complexes were then formed upon addition of CuBr, ZnI_2_ and [Rh(μ‐Cl)(cod)]_2_ (Scheme 7).

**Figure 4:**
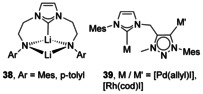
Mes=2,4,6‐Me_3_‐C_6_H_2_.

As a ligand‐transfer reagent for new Ta complexes, a homobimetallic Li‐NHC complex with two amido tethers was synthesised but could not be crystallised (Figure 4, **38**).^42^ A mesoionic carbene (MIC) has been tethered to an NHC to form an interesting hybrid bidentate ligand (**39**).^43^ They have different properties with the MIC being very strongly σ‐donating and thus has different binding modes and preferences for metal centres. Heterobimetallic complexes with Rh and Pd were obtained in high yields (83–92 %).^43^

### P‐Donor

**Figure 5:**
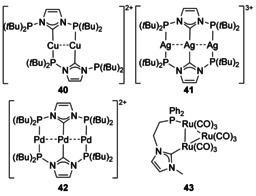
N‐Phosphanyl NHC complexes.

Phosphine donors attached directly to the NHC N atoms give rise to many multimetallic complexes, usually with Ag and Cu ions (for several examples, see Figure 5).^44^ Saturated NHCs with N‐phosphine substituents have also been developed and coordinated to Group 11 metal ions.^45^ Outside Group 11, only a handful of Pd complexes have also been structurally characterised (**42**).^46^ With a linker between the N atom and P donor, fewer multimetallic architectures are realised, and these are again mainly centred around Group 11 ions,44e, 47 A notable exception involves a phosphine‐tethered NHC bound to a Ru carbonyl cluster (**43**).^48^

### O And S‐donors

**Figure 6:**
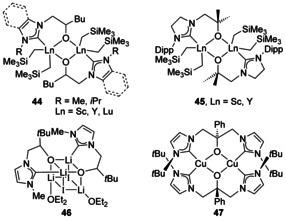
Alkoxide‐bridged species.

A common motif for bi‐ and multimetallic complexes formed from NHCs with O donors is a terminal NHC donor and a bridging oxygen donor, particularly for ‘hard’ metals (according to HSAB theory) (Figure 6, **44** and **45**).13a, 49 A homobimetallic Li dimer displayed similar bridging O atoms and an interesting cubic arrangement of Li/O/I atoms when co‐crystallised with LiI (**46**).^50^ A tridentate alkoxy‐NHC ligand generated the bimetallic Cu^I^ species **47**,^51^ with the hard–soft nature of the ligand appearing to stabilise the unusual square planar geometry for Cu^I^. Homobimetallic Pd and Ni complexes with bridging O donors have also been described,^52^ with heterobimetallic Ni/Li complexes formed when LiX by‐products are retained through coordination of Li to the O atoms.52a

**Figure 7:**
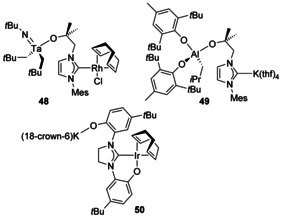
Bimetallic architectures with alkoxide/aryloxide tethers.

Several heterobimetallic compounds have been synthesised without bridging O atoms that give rise to different structures. A mixed Ta/Rh complex has been synthesised starting from either a Ta or Rh complex and by adding the other metal (Figure 7, **48**).^53^ Using the same NHC ligand, the heterobimetallic K/Al complex **49** has also been synthesised.^54^ Using a bis(aryloxide)tethered NHC, a heterobimetallic K/Ir complex has been crystallographically characterised (**50**).^55^ With sulfur donors, similar architectures are seen with bridging anionic S donors and terminal NHC ligands,^56^ and with neutral thioether tethers, bimetallic architectures can also be characterised.^57^

## Backbone Functionalisation with an Additional Donor

### C‐Donor

#### Cyclopentadienyl ligands

**Figure 8:**
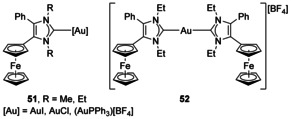
Ferrocene groups appended to NHC backbones.

Ferrocene has been appended onto an NHC backbone to produce multinuclear anti‐cancer drugs (Figure 8).^58^ It was found that the addition of a ferrocenyl group helped the production of reactive oxygen species due to the favourable reduction potential of the ferrocene groups. The ferrocene groups are relatively robust from a chemical point of view, allowing the NHC to be constructed in its presence, and were stable to the presence of base, methylating agents and silver and gold reagents.

#### Annulated π‐donors

**Figure 9:**
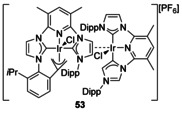
An Ir complex π‐bound to the double bond of an unsaturated NHC.

A complex has been characterised with Ir π‐bound to the double bond on an NHC backbone (Figure 9, **53**).^59^ This complex was synthesised as a by‐product in a relatively low yield but shows a fascinating mixture of carbene binding modes including an abnormal carbene, conventional carbene binding, activation of a Dipp (2,6‐diisopropyl‐phenyl) substituent as well as binding to the C=C double bond.

**Scheme 8:**
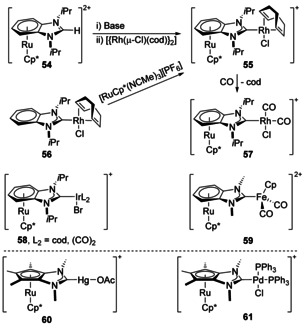
Benzannulated and cyclopentadienyl‐fused NHC complexes.

Complexation with larger π‐systems has also been achieved. Benzannulated NHCs have been synthesised with Ru coordinated to the benzannulated ring and with another metal fragment coordinated to the carbene (Scheme 8).^60^ This idea has also been extended to an NHC with pyrene annulation as well.^61^ NHCs can also include trimethylcyclopentadienyl‐fused to the NHC backbone with metals bonded to both the carbenic carbon and, with Ru, in a η^5^ coordination through the Cp ring annulated to the NHC backbone (**60** and **61**).^62^ This has so far not been extended to Fe.17c

#### NHC donor on backbone

**Scheme 9:**
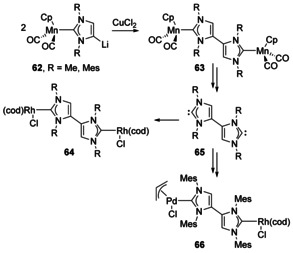
Ditopic dicarbene complexes.

Unsaturated NHCs bound to a [Mn(Cp)(CO)_2_] fragment can be deprotonated then coupled together using CuCl_2_ to give a ditopic ligand featuring two Mn centres (Scheme 9).^63^ These metal fragments can then be removed yielding the free dicarbene that was then coordinated to Rh and Pd fragments.^63^ Other types of bis(NHC)s that feature two diametrically opposed donors are also known, including ligands derived from benzannulation of both sides of a benzene backbone.^64^ Mesoionic di(1,2,3‐triazolylidene) ligands, which feature two 1,2,3‐triazolylidenes directly connected, have been show to bridge a Mn−Mn bond, and this bimetallic complex was shown to be an efficient catalyst for the oxidation of secondary alcohols and benzyl alcohol with *tert*‐butyl hydroperoxide.^65^

### Amine/N‐donor

**Figure 10:**
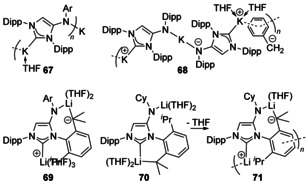
NHCs with anionic amido donors. Ar=4‐*t*BuC_6_H_4_.

An unsaturated NHC with a secondary aryl amine appended to the backbone was deprotonated to form a number of different architectures (Figure 10). With K[CH_2_Ph], monomeric or ion‐separated species were isolated, but multimetallic architectures were also structurally characterised as a coordination polymer (**67**) or with co‐crystallised K[CH_2_Ph] included (**68**).^66^

**Figure 11:**
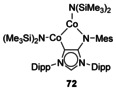
Dicobalt complex.

Using a similarly N‐functionalised NHC, reactions with alkyl lithiums saw the directed metalation of the isopropyl groups (Figure 10).^67^ Depending on the N‐substitution, the nature of the resulting species could be controlled. With a cyclohexyl substituent, cyclometallation occurs to the ‘normal’ NHC‐bound Li (**70**), although this can be further altered with the removal of the solvating THF leading to a dimeric structure featuring a η^6^ Li‐arene interaction between the NHC‐bound Li and the aryl ring (**71**). Using an aryl substituent, metalation occurs at the amido‐bound Li group instead (**69**). With Co, an abnormal carbene was formed as a dinuclear cobalt species with a Co−Co metal bond (Figure 11, **72**).^68^ This fragment was supported between the abnormal carbene and the amide functionality and was sterically protected by the bulky N(SiMe_3_)_2_ groups. The large steric bulk on the carbene fragment also helps with kinetic stabilisation.^69^

### Phosphine donor

**Figure 12:**
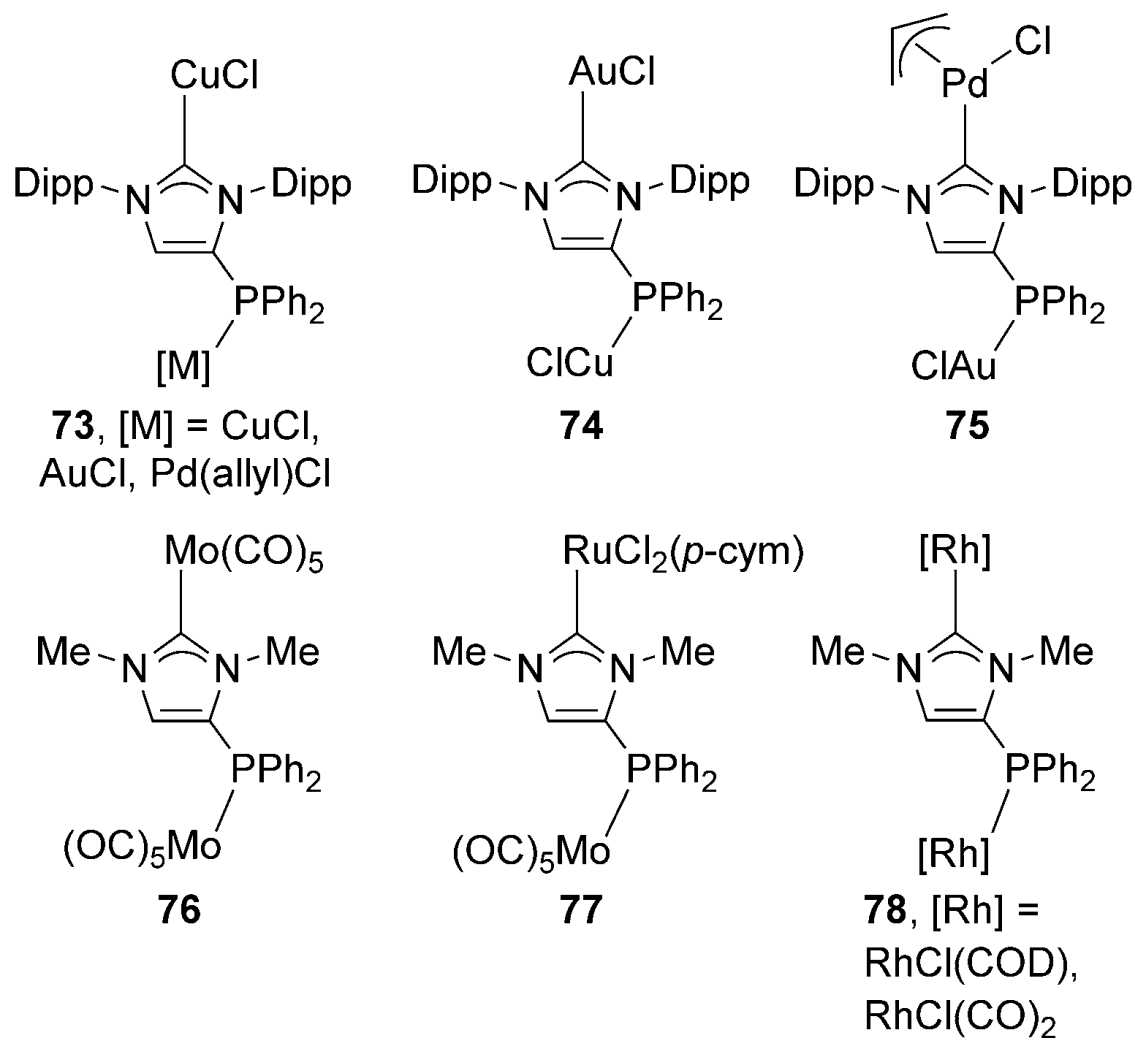
Bimetallic complexes with ditopic phosphine‐NHC ligands.

Combining a phosphine donor on the NHC backbone with the strongly σ‐donating NHC moiety is an appealing prospect. A PPh_2_ group can be appended onto the backbone of a unsaturated NHC,^70^ and this bifunctional ligand was then shown to coordinate to a variety of Cu, Au and Pd metal fragments to the two different donor positions (Figure 12).^71^ Additional work has been performed with N‐Me substituted phosphinocarbenes as well (**76**–**78**).^72^

**Scheme 10:**
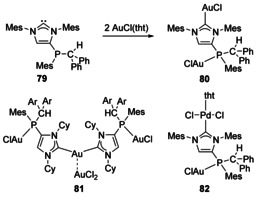
Complexation of phosphino‐NHCs formed from phosphaalkenes. tht=tetrahydrothiophene; Ar=4‐F‐C_6_H_4_.

In 2009, Gates and co‐workers discovered ‘abnormal’ reactions of phosphaalkenes with NHCs.^73^ This offered a route to NHCs functionalised on the backbone with a phosphine group and coordination of two equivalents of AuCl was demonstrated (Scheme 10, **80**).^73^ This has been extended to other phosphaalkenes^74^ as Au and Pd complexes (**81** and **82**).^75^ This route is particularly interesting as the phosphines generated are stereogenic due to the presence of three different substituents, thus leading to the potential of chiral ligands if enantioselective routes could be developed.

**Figure 13:**
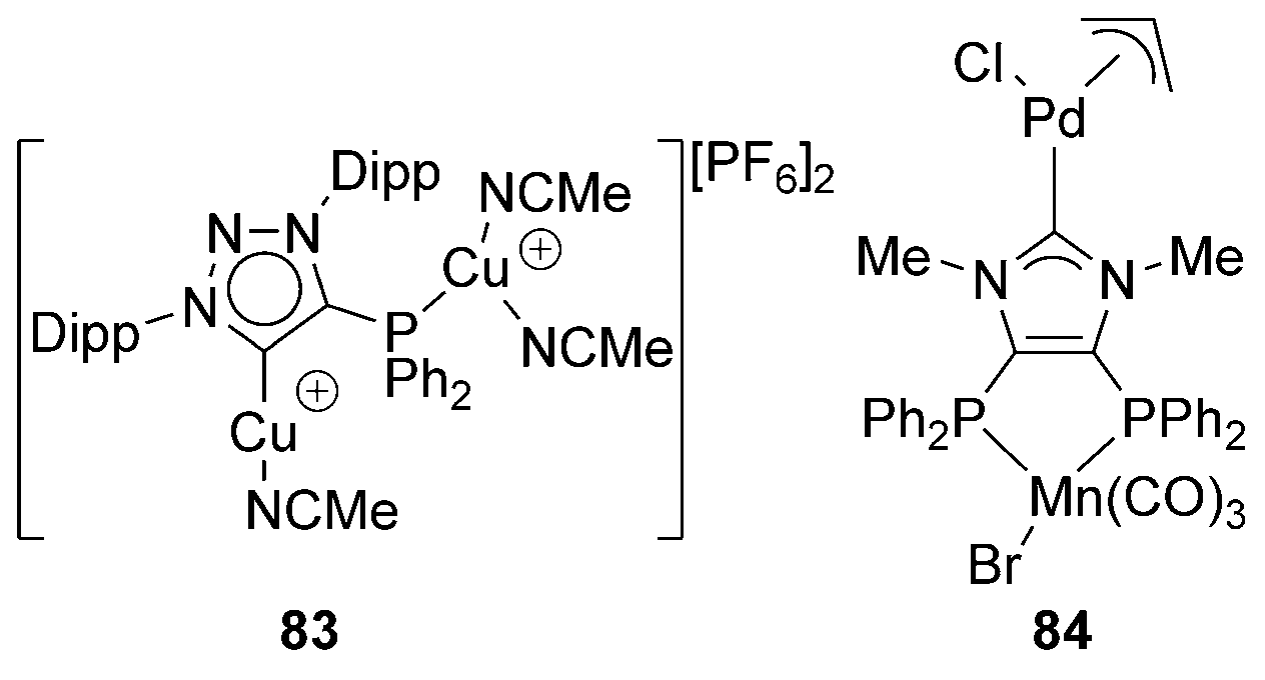
Bimetallic complexes with additional phosphine donors.

1,2,3‐Triazol‐5‐ylidenes are carbenes that feature three N atoms in the heterocyclic ring. Appending a PPh_2_ group next to the carbenic C atom afforded a variety of dinuclear (and trinuclear for Au) complexes with Cu, Ag and Au (Figure 13, **83**).^76^ An NHC with two PPh_2_ donors on the backbone has been synthesised, and a Mn/Pd heterobimetallic complex was structurally characterised (**84**).^77^

### O‐Donor

**Scheme 11:**
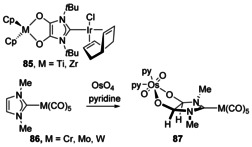
Bimetallic NHC complexes featuring O donors on the backbone.

Complexes with a chelating dianionic bis(O‐donor) ligand have been used to bind ‘hard’ Group 4 metals to the O donors and ‘soft’ Ir to the NHC (Scheme 11, **85**).^78^ The synthesis started with the Ir complex of a diketo‐NHC, and reaction with Ti^II^/Zr^II^ reagents caused the formation of an unsaturated dialkoxide NHC with M^IV^ coordinated. The only saturated NHC with O donors attached to the backbone was synthesised by addition of OsO_4_ to the unsaturated NHC complex **86** forming the chelating, O‐bound Os complex **87** that retains the M(CO)_5_ fragment bound to the carbenic C atom.^79^ This highlights the relative robustness of the key NCN fragment whereas the backbone of the NHC reacts, allowing for in situ metalation of the backbone without the loss of coordination from the carbene.

**Scheme 12:**
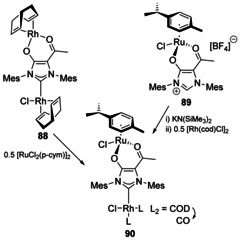
NHC‐acac ditopic complexes.

Fusing the acetylacetonate (acac) moiety with an NHC backbone gives a bifunctional NHC with an X,L pocket in addition to the typical carbene L donor site (Scheme 12).^80^ Although the Ru−Rh bimetallic NHC complex **90** was synthesised by sequential metalation with [RuCl_2_(*p*‐cym)]_2_ (yielding **89**) and then [Rh(cod)Cl]_2_, it was observed that reacting the dirhodium complex **88** with [RuCl_2_(*p*‐cym)]_2_ also led to the Ru−Rh complex **90** regenerating [Rh(cod)Cl]_2_. This highlights the lability of the acac moiety bound to late transition metals compared to the carbene fragment.

**Scheme 13:**
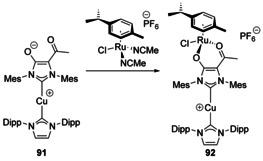
NHC‐acac ditopic complexes with Cu.

A bimetallic bis‐NHC complex with Cu and Ru (**92**) was also synthesised with an acac‐NHC ligand (Scheme 13). The NHC pocket was favoured by the Cu centre whereas the acac coordinated to Ru. This preference was also observed in the synthesis of the mono‐Cu complex (**91**) in which, upon diprotonation to give the carbene, rearrangement from κ^2^‐O,O to carbene binding occurred.^81^

**Figure 14:**
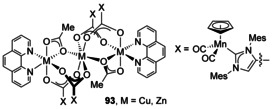
Multi‐metallic clusters using carboxylate donors.

After lithiation at the backbone and reaction with CO_2_, a Mn complex of an NHC with a carboxylic acid on the backbone was synthesised. Two complexes with Cu and Zn fragments were then synthesised (Figure 14, **93**).^82^

## Backbone Metalated

NHCs usually feature the divalent carbon at the 2‐position so that stabilisation from both N‐atoms is present. However, ‘abnormal’ carbenes^83^ feature the divalent carbon at the 4‐ or 5‐position on the backbone, yielding stronger σ‐donor ligands.^84^ This backbone metalation produces a mesionic compound, but subsequent deprotonation of the 2‐position would lead to a formally anionic, ditopic NHC able to assemble bi‐ and multimetallic architectures.^85^ 1,2,4‐Triazolyl‐3,5‐di‐ylidene ligands contain three N atoms and two divalent carbene atoms, leading to an extensive bimetallic coordination chemistry.^86^

### Groups 1, 2 and 12

**Figure 15:**
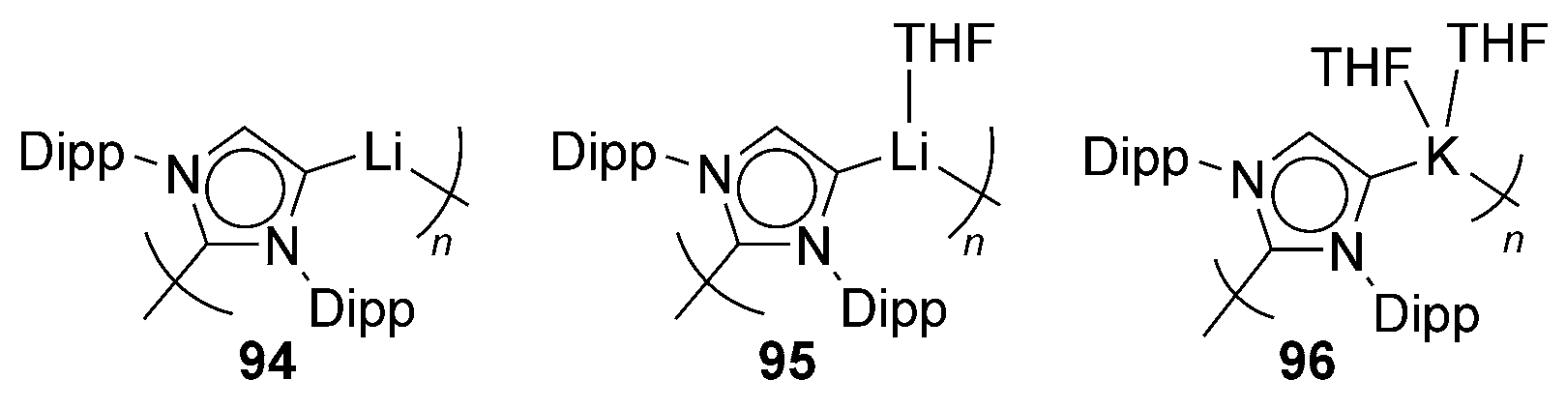
Deprotonation of IPr yielding coordination polymers.

1,3‐Bis(2,6‐diisopropylphenyl)imidazol‐2‐ylidene (IPr) was shown to react with *n*BuLi through deprotonation of the backbone forming a polymeric structure featuring coordination from both normal and abnormal NHC motifs (**94**, **95**, Figure 15).^87^ The potassium analogue has also been described (**96**),^88^ and was formed through transmetalation of LiIPr with KO*t*Bu. Attempts to remove the K ion with the addition of 2,2,2‐cryptand led to protonation of the carbene and degradation of the cryptand, suggesting the unstable nature of the free anionic NHC.

**Figure 16:**
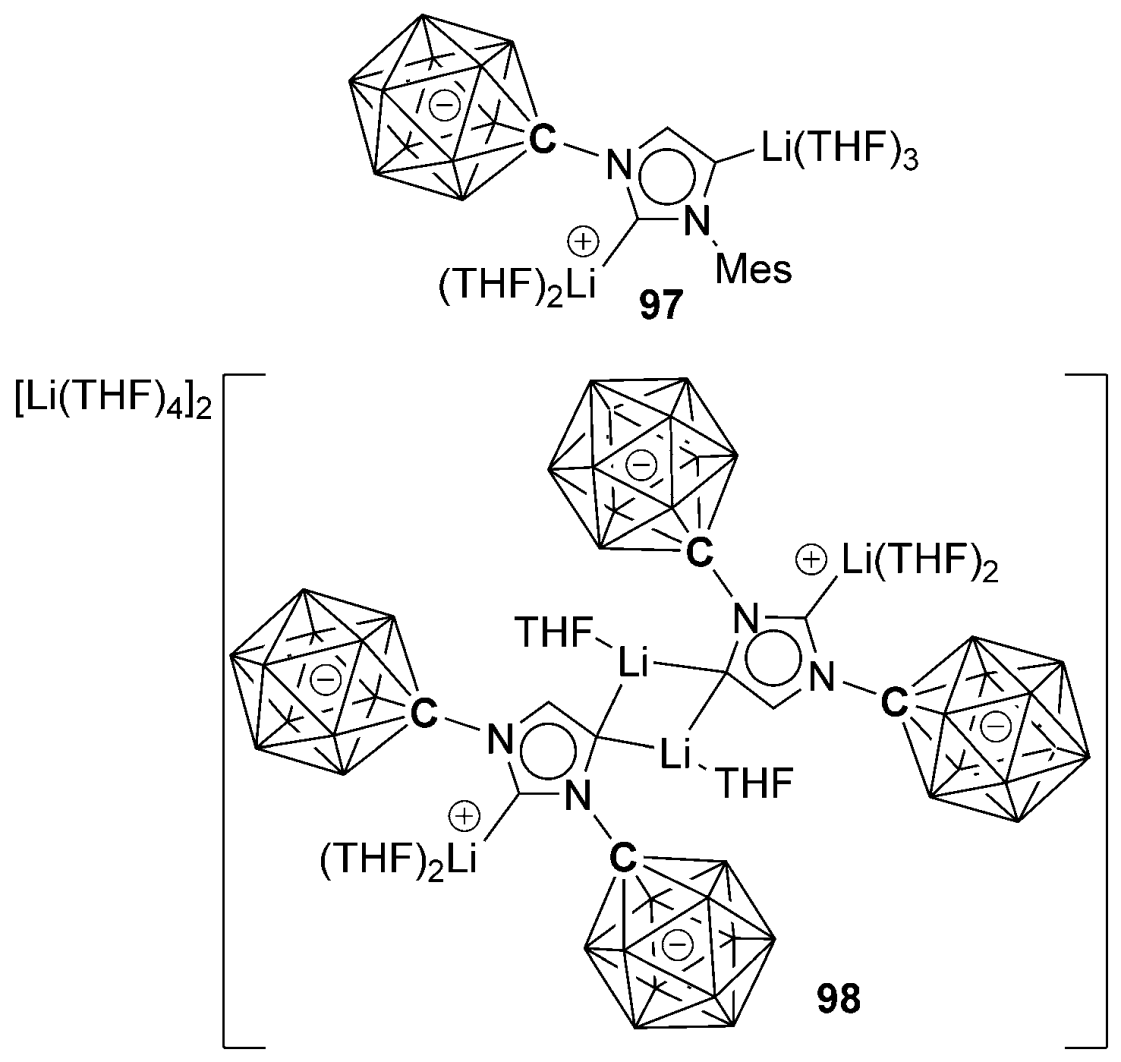
Carborane vertices are B atoms unless otherwise stated.

Using the anionic N‐carboranyl fragment [CB_11_H_12_]^−^ produced a similar outcome but with two Li atoms per NHC, yielding a molecular species (Figure 16, **97**).^89^ An analogous NHC with two N‐carboranyl substituents has also been synthesised, and was used to generate a multimetallic species (**98**).^90^

**Figure 17:**
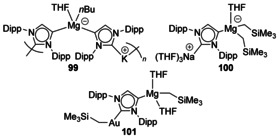
Magnesiated IPr complexes.

A variety of species that feature magnesiated carbenes bound to Group 1 cations have recently been structurally characterised. Synergic deprotonation of IPr with [{KMg(TMP)_2_(*n*Bu)}_6_] (TMP=2,2,6,6‐tetramethylpiperidide) yielded a structure with magnesium attached to the backbones of two NHCs with potassium bound to the traditional C2 carbenic carbon (Figure 17, **99**).^91^ Using analogous sodium reagents yielded either a structure with Mg attached to three NHC backbones or ‘templated metalation’ of the Dipp group in addition to deprotonation of the NHC backbone.^91^ Simpler, monomeric Mg/Na species were achieved using stepwise addition of reagents (**100**).^92^ The addition of [AuCl(SMe_2_)] with loss of NaCl revealed transfer of an R group (R=CH_2_SiMe_3_) from Mg to Au forming a heterobimetallic complex (**101**).^92^

**Figure 18:**
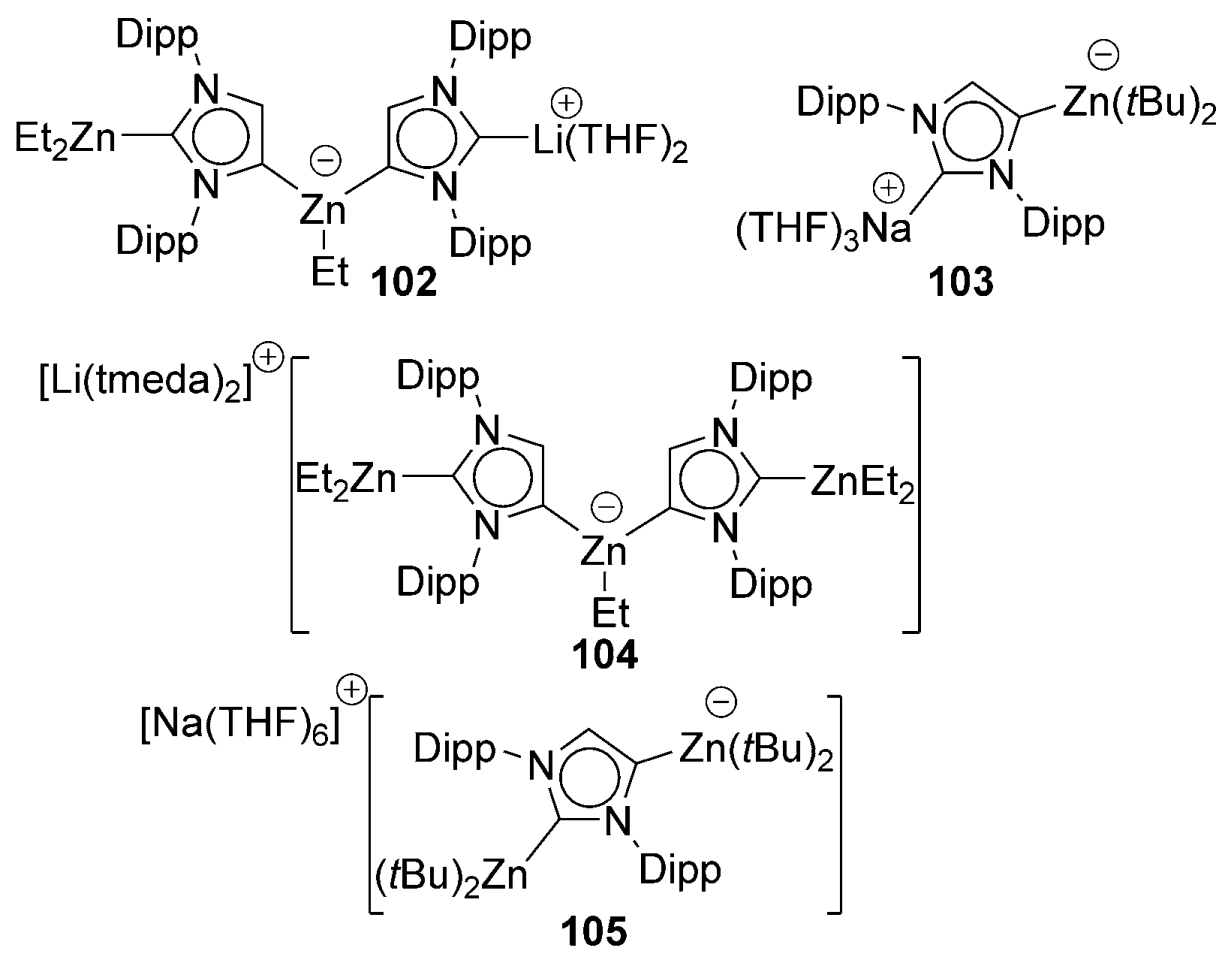
Zincate structures based on IPr. tmeda=Me_2_NC_2_H_4_NMe_2_.

A series of triorganozincates using backbone‐metaled NHCs have been described. The C2 position is coordinated to either a group 1 cations or a dialkyl zinc (Figure 18).^93^

### Group 1 + p‐block

**Figure 19:**
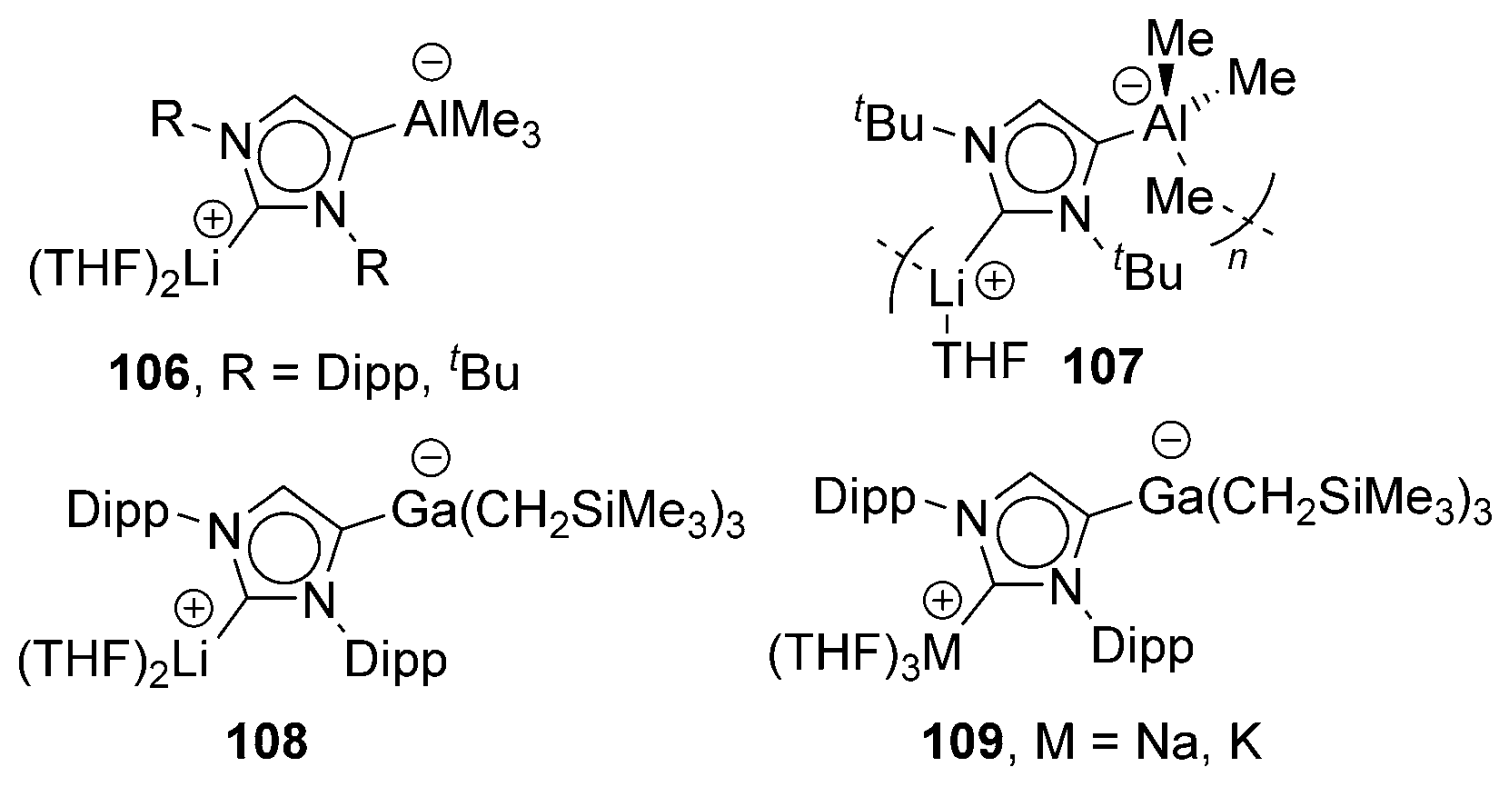
Group 13/ alkali‐metal complexes.

Addition of AlMe_3_ to deprotonated IPr gave coordination of AlMe_3_ at the 4‐position (Figure 19, **106**),^87^ and the same motif was also achieved by addition of *n*BuLi to an AlMe_3_‐coordinated abnormal carbene (**107**).^94^ The coordination of the p‐block element to the NHC backbone is proposed to be driven by the carbophilic nature of the p‐block elements in comparison to the s‐block.85b Similar preference for Ga to bind to the backbone has also been observed (**108** and **109**).^95^

**Figure 20:**
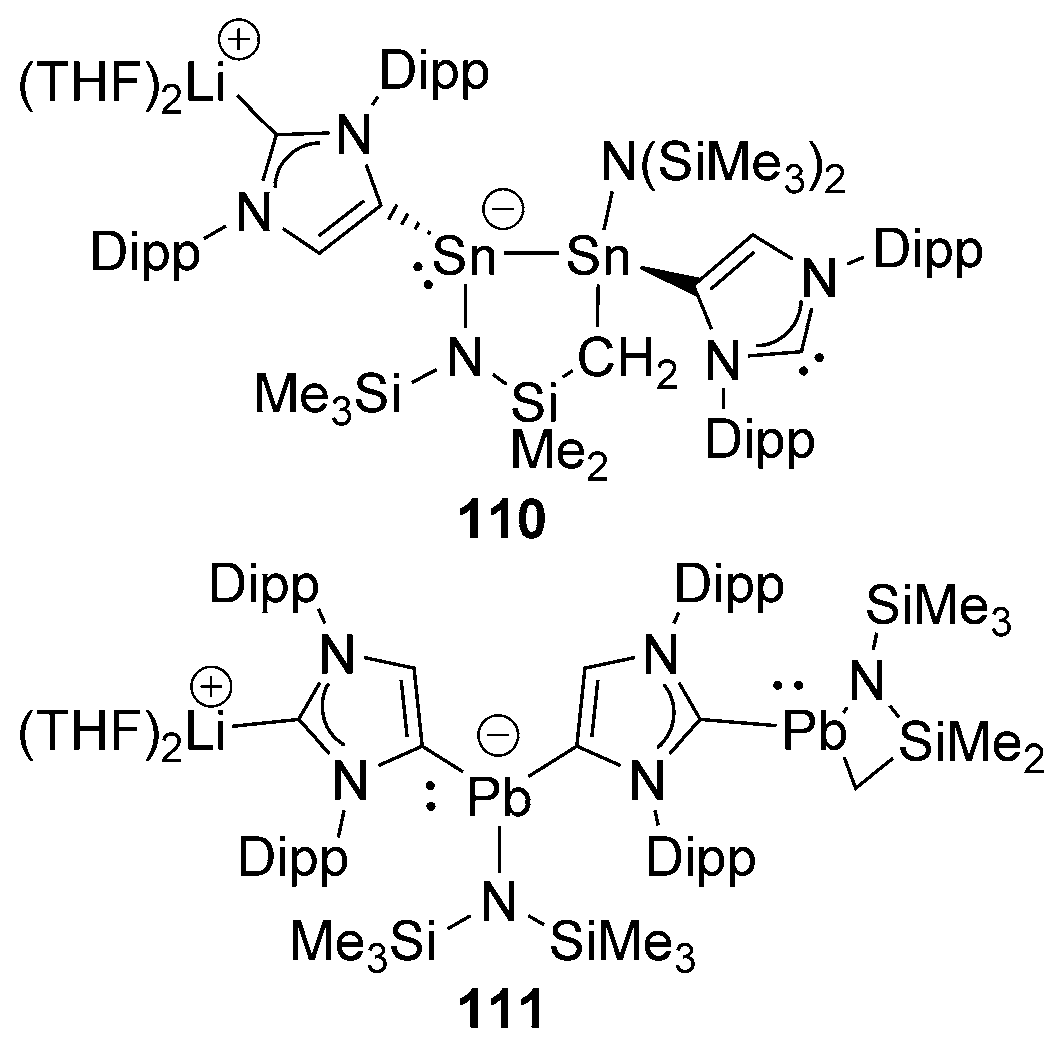
Group 14/ alkali‐metal complexes.

Reactions between a backbone‐deprotonated carbene and either [Sn{N(SiMe_3_)_2_}_2_] or [Pb{N(SiMe_3_)_2_}_2_] led to a series of reactions culminating in some unusual multimetallic products (Figure 20).^96^ With Sn, a distannane was formed supported by a cyclometalated hexamethyldisilazide (HMDS) ligand (**110**), whereas the final crystallographically characterised Pb species had a Pb atom linking two backbone‐deprotonated carbenes with one NHC bound to a lithium cation and the other to a Pb^II^ fragment chelated by a cyclometalated HMDS ligand (**111**).^96^

### Group 1 + transition metals

**Figure 21:**
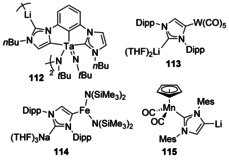
Transition‐metal/ alkali‐metal complexes.

Reaction of a Ta bis(carbene)phenyl pincer complex with excess lithium *tert*‐butylamide led to an interesting bimetallic product, although its only characterisation was by single‐crystal X‐ray diffraction (Figure 21, **112**). One of the NHC side arms had been deprotonated and subsequently rearranged to form an abnormal binding mode to Ta with Li binding at the C2 position. This molecule then aggregated into a dimer.^97^ The reaction of backbone‐deprotonated IPr with [W(CO)_5_(THF)] led to the coordination of W at the 4‐position with Li coordinated at the 2‐position (**113**).^98^ Likewise, the deprotonation of IPr with NaCH_2_SiMe_3_ followed by the addition of [Fe{N(SiMe_3_)_2_}_2_] led to coordination of the Fe at the 4‐postion with Na coordinated at the 2‐position (**114**).^99^ In contrast, IMes (1,3‐bis(2,4,6‐trimethylphenyl)‐imidazol‐2‐ylidene) bound to a [Mn(Cp)(CO)_2_] fragment was directly metalated using *n*BuLi retaining [MnCp(CO)_2_] at the 2‐position and binding Li at the 4‐position (**115**).^82^

**Figure 22:**
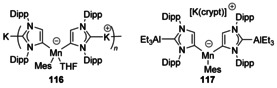
Manganese complexes from reduction.

Chemical reduction of [Mn(Mes)_2_(IPr)] with KC_8_ was found to give the first example of a transition‐metal complex containing an anionic N‐heterocyclic dicarbene ligand. The products that resulted (Figure 22) featured loss of one mesityl group and deprotonation of the NHC backbone yielding complexes with Mn coordinated at the 4‐position to two abnormal carbenes. The ‘normal’ two position was then coordinated to K as a coordination polymer, or, upon addition of AlEt_3_ and cryptand, with AlEt_3_ coordinated instead.^100^

### Group 1+Ln

**Figure 23:**
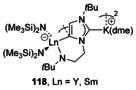
Dimeric complex featuring rare earth and potassium ions.

Dimeric potassium‐lanthanide complexes were synthesised from reduction of the starting precursor with K(naphthalide). The NHC switched from 2‐coordination to the Ln to K, with the Ln now being coordinated between the abnormal carbene and the amide tether. In addition, the K cations also interact with the abnormal carbenes giving rise to a dimeric structure (Figure 23).^101^

### p‐Block only

**Scheme 14:**
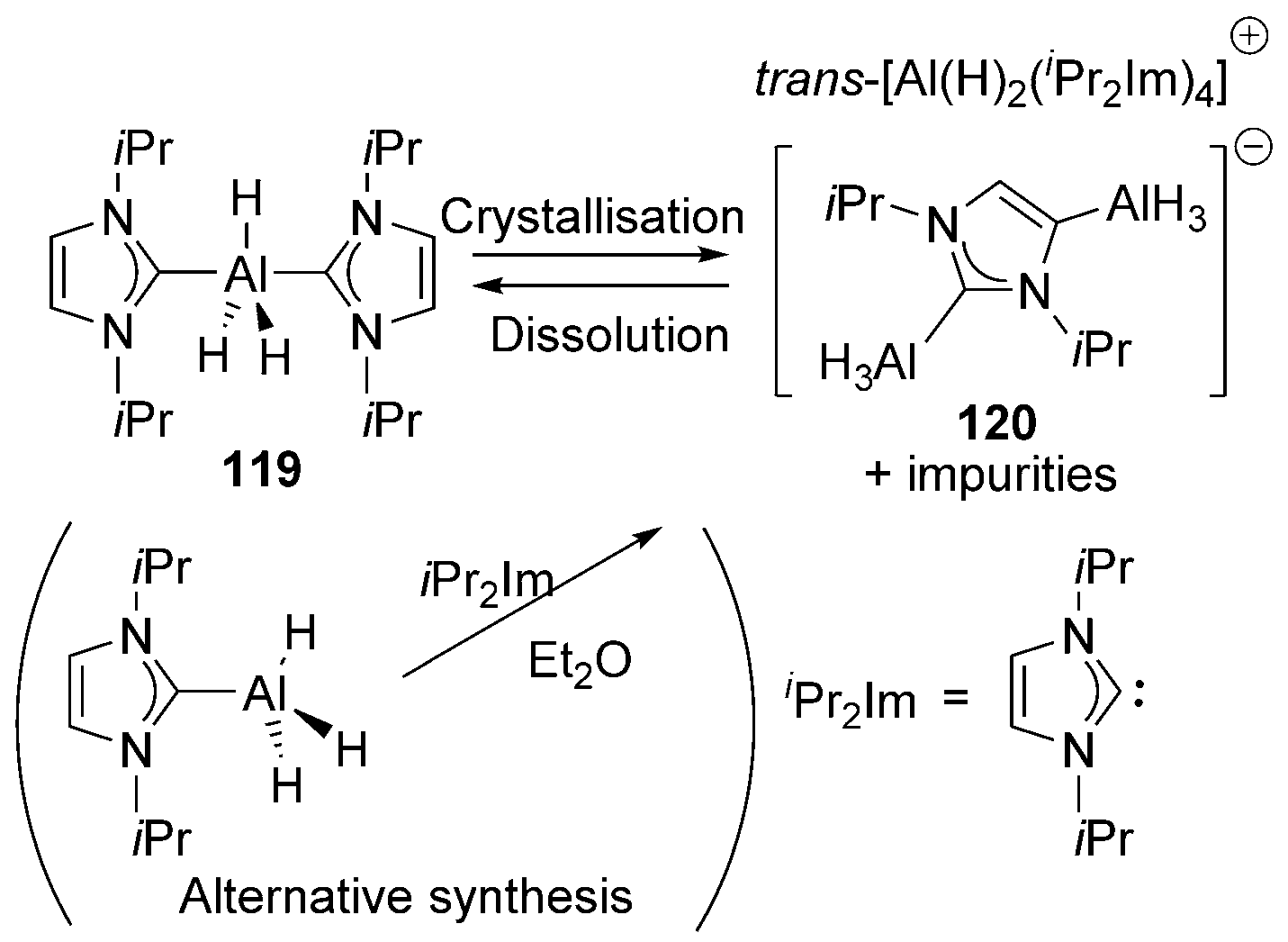
Interconversion of NHC‐alane complexes.

With alane (AlH_3_), addition of two equivalents of IPr formed a bis‐coordination complex which existed in equilibrium with ligand redistribution products (Scheme 14). In the ionised product **120**, the cation is [Al(H)_2_(IPr)_4_]^+^ whereas the anion contains AlH_3_ bound to both the 2‐ and 4‐postions of a backbone metalated carbene.^102^ Alane is a strong reducing agent thus the NHC remaining intact is an indication of the robustness of the carbene donor part as well as the flexibility of metalation at the backbone in the formation of the abnormal carbene.

### Transition‐metal only

**Scheme 15:**
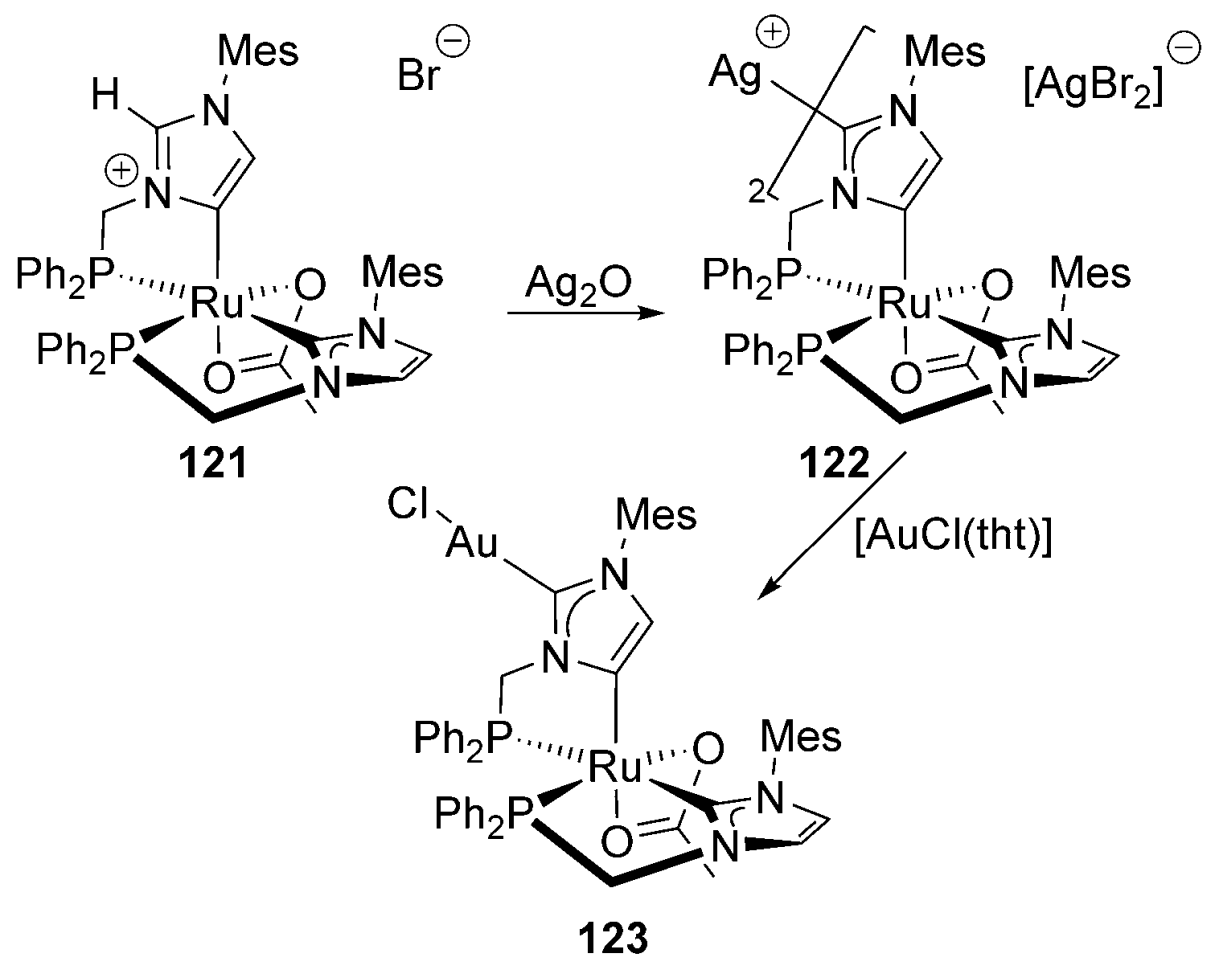
Bimetallic complexes of Ru and Group 11.

Starting from a Ru complex with a phosphine‐tethered abnormal NHC ligand, reaction with Ag_2_O afforded a heterobimetallic Ag/Ru complex with Ag bound at the 2‐position (Scheme 15, **122**).^103^ This was then transmetalated with AuCl(tht) (tht=tetrahydrothiophene) to afford the analogous Ru/Au heterobimetallic complex (**123**).

**Figure 24:**
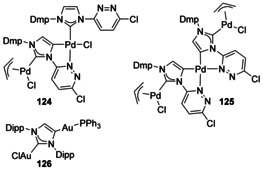
Homometallic Pd and Au complexes.

Backbone‐metalated Pd complexes have been synthesised using an asymmetric NHC ligand with Pd(allyl)Cl coordinated at the C2 positions (Figure 24, **124** and **125**).^104^ A homobimetallic Au complex has also been synthesised from transmetalation of the Zn/Na anionic dicarbene **103** with two equivalents of [AuCl(PPh_3_)] leading to AuCl coordinated at the 2‐position and Au(PPh_3_) at the 4‐position (**126**).93b

**Scheme 16:**
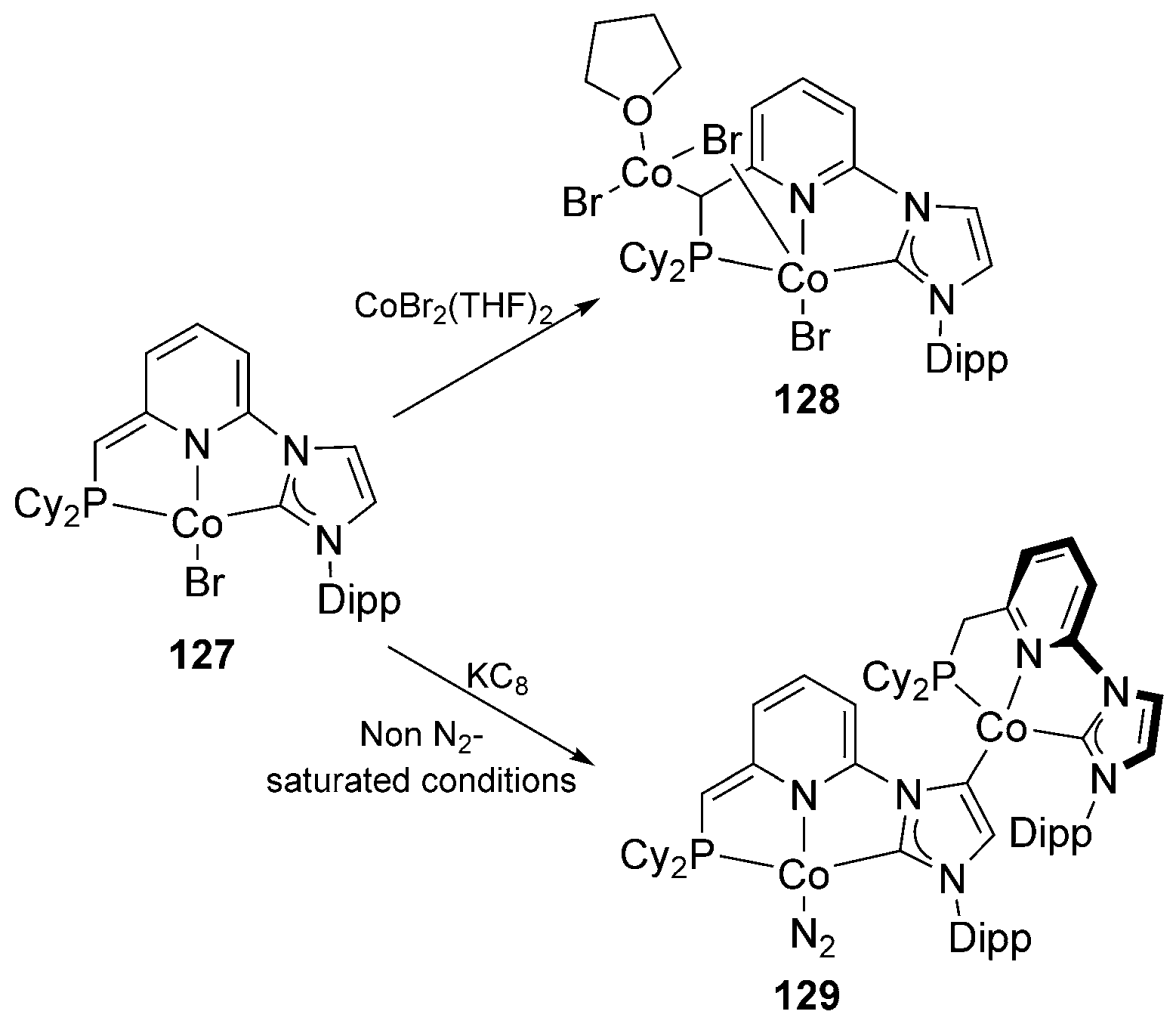
Dicobalt complexes with an NHC pincer ligand.

The benzylic position between phosphine and pyridyl substituents in phosphino‐picolines is acidic and thus can be metalated, which has been observed for a number of related compounds.^105^ The dearomatised phosphino‐picoline NHC‐Co pincer complex **127** 106 was found to react with CoBr_2_(THF)_2_ to give a bromide bridged dicobalt complex (Scheme 16, **128**). Reduction of **127** with KC_8_ in the presence of N_2_‐saturated solvents gave the straightforward N_2_ complex, whereas in the absence of N_2_‐saturated conditions formed the backbone‐metalated dicobalt complex **129**. This is presumably due to a lack of N_2_ to stabilise the reactive Co^I^ centre.

## Summary and Outlook

It has been shown that a wide area of coordination chemistry has developed in which NHC ligands act to bind two or more metals. Common synthetic pathways to these complexes have been described, helping to bring together disparate literature in the fields of organometallic chemistry of the transition metals, main‐group metals and the rare earths. Understanding the origin of these reactions has the potential to not only improve the synthesis of multimetallic architectures, but also to help inform chemists about decomposition routes for NHC complexes, helping to guide designs for next‐generation organometallic complexes and catalysts. Examples of this are the formation of [C,N]‐bonded bridging NHC ligands through N−C (or N−P for N′‐phosphanyl NHCs) bond activation, that could be an important decomposition pathway for low oxidation state and low‐coordinate transition‐metal complexes and intermediates. This sets up a striking analogy to phosphines for which P−C bond cleavage is also considered to be an important decomposition route.^107^ Another example is the propensity of unsaturated NHC ligands to undergo backbone diprotonation forming abnormal or mesionic carbene ligands, which can be mitigated by the implementation of saturated NHC ligands. As a counterpoint to this, examples have been shown of the exceptional stability of the metal–carbene interaction, in which a wide variety of reactions can occur to the backbone or substituents without cleaving the M−C bond. This underlies the importance of NHC ligands in homogeneous catalysis as well, where the NHC can function as a very competent spectator ligand. The field of NHC ligands in bimetallic architectures is likely to expand further due to increasing access to analytical equipment that can identify large complexes and clusters built up from functionalised carbene ligands. X‐Ray crystallography has proven to be vital in analysing these often highly asymmetric complexes, which can also be present only in small quantities as by‐products in reaction mixtures, together with new methods and better equipment in mass spectrometry and NMR spectroscopy. Areas that look particularly interesting for exploitation include coordination of metals other than Ru to benzannulated π‐donors (phenyl and Cp rings), including Fe that would lead to redox control over the electronic properties of the NHC ring. There is also likely to be a more extensive coordination chemistry of the π‐bond in simple unsaturated NHCs waiting to be discovered as well. It is also clear that that many bimetallic species with functionalised NHC Ligands have great potential in the field of cooperative catalysis, although at the moment this remains seriously underexplored. Overall, we hope that the specific identification of NHCs in the formation of bimetallic architectures should help promote this emerging area and encourage further ligand development to either exploit or block these reaction pathways, as required.

## Conflict of interest

The authors declare no conflict of interest.

## Biographical Information

*Kieren Evans graduated with a M.Sc. degree from the University of York in 2015 and worked in the group of Prof. Ian Fairlamb on Au‐catalysed reactions applied to organic synthesis. He is currently a PhD student at Heriot‐Watt University under the supervision of Dr Stephen Mansell. His research interests are on developing functionalised NHC ligands for use in transition‐metal catalysis*, *with a particular focus on C−H activation reactions*.

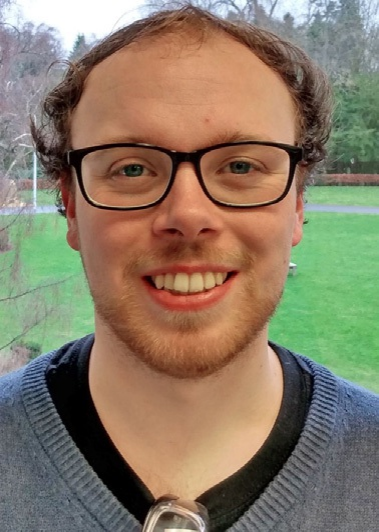

## Biographical Information

*Stephen Mansell is an Assistant Professor in the Institute of Chemical Sciences at Heriot‐Watt University. His research interests focus on main‐group and transition‐metal chemistry applied to catalysis through the design and use of unusual ligands including phosphino‐phosphinines and tethered NHCs. He obtained his MSci degree from Imperial College London in 2005 and his PhD from The University of Bristol in 2009. After post‐doctoral work in boron chemistry and small‐molecule activation mediated by uranium complexes, he started his current position in 2013*.